# Garlic Origin Traceability and Identification Based on Fusion of Multi-Source Heterogeneous Spectral Information

**DOI:** 10.3390/foods13071016

**Published:** 2024-03-26

**Authors:** Hao Han, Ruyi Sha, Jing Dai, Zhenzhen Wang, Jianwei Mao, Min Cai

**Affiliations:** 1 School of Biological and Chemical Engineering, Zhejiang University of Science and Technology, Hangzhou 310023, China; 212103817026@zust.edu.cn (H.H.); nanodoudou123@163.com (J.D.); cnhkwzz@163.com (Z.W.); zjhzmjw@163.com (J.M.); mincai@zjut.edu.cn (M.C.); 2 Zhejiang Provincial Key Laboratory for Chemical & Biological Processing Technology of Farm Product, Hangzhou 310023, China

**Keywords:** ultraviolet spectroscopy, mid-infrared spectrum, machine learning, origin traceability

## Abstract

The chemical composition and nutritional content of garlic are greatly impacted by its production location, leading to distinct flavor profiles and functional properties among garlic varieties from diverse origins. Consequently, these variations determine the preference and acceptance among diverse consumer groups. In this study, purple-skinned garlic samples were collected from five regions in China: Yunnan, Shandong, Henan, Anhui, and Jiangsu Provinces. Mid-infrared spectroscopy and ultraviolet spectroscopy were utilized to analyze the components of garlic cells. Three preprocessing methods, including Multiple Scattering Correction (MSC), Savitzky–Golay Smoothing (SG Smoothing), and Standard Normalized Variate (SNV), were applied to reduce the background noise of spectroscopy data. Following variable feature extraction by Genetic Algorithm (GA), a variety of machine learning algorithms, including XGboost, Support Vector Classification (SVC), Random Forest (RF), and Artificial Neural Network (ANN), were used according to the fusion of spectral data to obtain the best processing results. The results showed that the best-performing model for ultraviolet spectroscopy data was SNV-GA-ANN, with an accuracy of 99.73%. The best-performing model for mid-infrared spectroscopy data was SNV-GA-RF, with an accuracy of 97.34%. After the fusion of ultraviolet and mid-infrared spectroscopy data, the SNV-GA-SVC, SNV-GA-RF, SNV-GA-ANN, and SNV-GA-XGboost models achieved 100% accuracy in both training and test sets. Although there were some differences in the accuracy of the four models under different preprocessing methods, the fusion of ultraviolet and mid-infrared spectroscopy data yielded the best outcomes, with an accuracy of 100%. Overall, the combination of ultraviolet and mid-infrared spectroscopy data fusion and chemometrics established in this study provides a theoretical foundation for identifying the origin of garlic, as well as that of other agricultural products.

## 1. Introduction

China is the world’s largest producer of garlic, responsible for over 60% of the global planting area. This production is mainly concentrated in provinces such as Shandong, Jiangsu, Anhui, Henan, and Yunnan. China’s garlic production contributes to over 70% of the global total [1]. Garlic (*Allium sativum*) is a perennial herb of the Liliaceae family that is commonly used as a food seasoning. It is renowned for its nutritional and medicinal benefits, including antibacterial [2], anticancer [3], hypolipidemic [4], anti-inflammatory [5], and antioxidant effects [6].

The chemical and nutrient compositions of garlic can be influenced by various geographical factors. Different varieties of garlic grown in distinct regions may exhibit significant variations in flavor and other functional attributes, which can impact consumer preferences and acceptance levels [7,8]. To promote brand recognition and foster the sustainable development of the garlic industry, it is important to establish geographic indicators for garlic agricultural products. Agricultural product traceability has gained attention from researchers as a means to effectively differentiate products of diverse origins. For instance, Biancolillo et al. [9] employed headspace solid-phase microextraction (HS-SPME)-gas chromatography-mass spectrometry (GC-MS) to analyze 68 red garlic samples from four regions in Italy, successfully distinguishing their origins. Similarly, Mi et al. [10] utilized targeted multi-elemental analysis, non-targeted volatile analysis, and metabolomics to establish chemical fingerprints of garlic from Langfang, Kaifeng, Jining, and Dali in China, enabling garlic origin verification.

Conventional methods utilized for tracing the origin of garlic, including mass spectrometry, isotopic analysis, and nuclear magnetic resonance, face obstacles due to intricate pre-treatment procedures and exorbitant operational expenses. This underscores the urgent need for the advancement of swift, non-destructive, and uncomplicated identification techniques. Spectroscopic technologies offer the advantages of simplicity, minimal or no sample pretreatment, and low sample damage [11]. Infrared, ultraviolet, and other spectroscopic techniques are now widely employed for the origin and species identification of agricultural products and traditional Chinese medicine. Pan et al. [12] collected the near-infrared reflectance spectra of orange peel storage age and subjected these data to Savitzky–Golay convolutional smoothing, first-order derivatives (SGFD), and SNV. Then, three discriminant models were established based on the preprocessed data, namely, RF, K Nearest Neighbor (KNN), and Linear Discriminant Analysis (LDA). The accuracy for origin recognition was 96.99%. The identification accuracy for the storage age of Guangdong orange peel was 100%, while for Sichuan orange peel, it was 97.15%. This highlights that NIRS combined with machine learning enables rapid and simultaneous identification of the origin and orange peel storage age in the field. Tong et al. [13] established a rapid identification method for the origin of rice based on Fourier transform near-infrared spectroscopy using a combination of Principal Component Analysis (PCA) and Deep Learning (DL) techniques. The results showed that the total recognition accuracy for sample calibration and testing using the PCA method reached 91.04% and 87.10%, respectively, while the DL method achieved 100% accuracy in both cases.

In certain situations, test samples of different kinds may not have enough spectral information to fully represent their chemical characteristics. This requires data fusion from various sources, which integrates information from different sources to enhance the accuracy of decision models by reducing the risk of missing characteristic variables present in single datasets. Data fusion is divided into three categories: Low-Level Data Fusion (LLDF), Mid-Level Data Fusion (MLDF), and High-Level Data Fusion (HLDF) [14,15]. He et al. [16] studied liquor spectral properties using ultraviolet spectroscopy, near-infrared spectroscopy, and multidirectional fluorescence spectroscopy, achieving high accuracy in identifying different liquors compared to single-spectrum analysis. Luan et al. [17] conducted rice origin identification using near-infrared, mid-infrared, and Raman spectra, showing improved recognition accuracy with data fusion compared to single-spectrum models.

In today’s digital age, Traceability 4.0 has become a crucial concept in manufacturing and managing supply chains [18]. It stresses the need for complete traceability of product production and flow processes through the use of digital technologies and data-driven approaches. Industry 4.0 technologies such as the Internet of Things (IoT), big data analytics, blockchain, and artificial intelligence are extensively utilized to verify the origin of products and resolve other traceability issues. For example, manufacturers can use IoT sensors and blockchain technology to monitor the transportation and processing stages of raw materials and products throughout the entire supply chain, ensuring the traceability of product origins and guaranteeing their quality and safety. By integrating these digital technologies, new possibilities for achieving product traceability emerge, promoting greater trust and transparency among consumers.

This study aimed at developing a rapid, dependable, and straightforward method for tracing the origin of garlic samples sourced from five distinct regions in China, namely Shandong, Jiangsu, Anhui, Henan, and Yunnan Provinces. The garlic samples were analyzed using ultraviolet and mid-infrared spectroscopy, and the collected spectral data were preprocessed to eliminate any nonlinear perturbations and random noises. The raw data were then subjected to feature extraction using GA, and the single spectral information was evaluated using LLDF. The ultimate goal of this study was to provide a rapid identification method for selecting raw materials for garlic export geographic indications.

## 2. Materials and Methods

### 2.1. Sample Material

**Figure 1:**
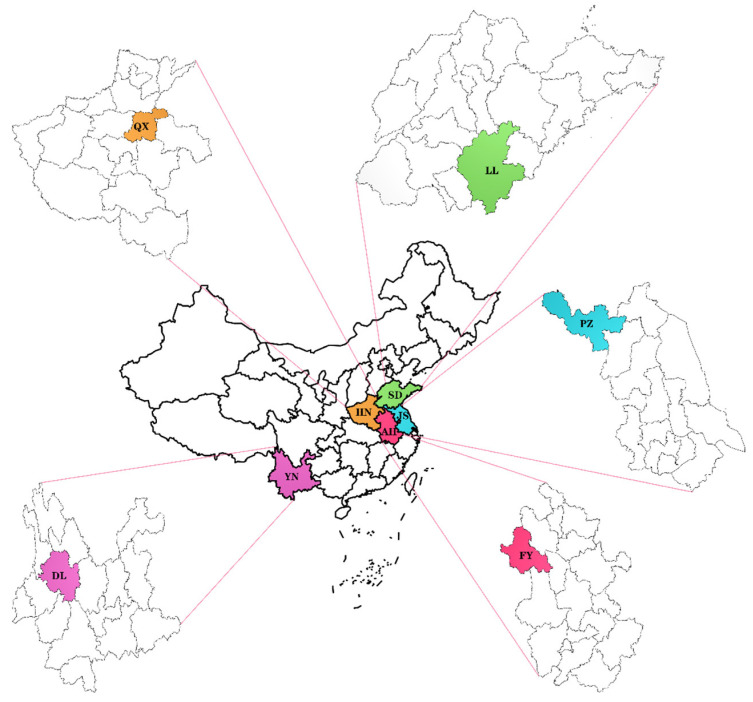
Regional distribution of garlic samples.

This study collected a total of 225 samples of purple-skinned garlic from five different regions in China. As shown in Figure 1. The regions included Lanling, Shandong Province (LL, SD); Dali, Yunnan Province (DL, YN); Fuyang, Anhui Province (FY, AH); Qixian, Henan Province (QX, HN); and Pizhou, Jiangsu Province (PZ, JS). The samples were numbered 1–45 for Lanling, Shandong; 46–91 for Dali, Yunnan; 92–139 for Fuyang, Anhui; 140–183 for Qixian, Henan; and 184–225 for Pizhou, Jiangsu.

### 2.2. Spectra Acquisition

A Fourier transform infrared spectrometer, Bruker VERTEX 70 from Germany, was used to conduct the testing. The scanning range was set to 400~4000 cm^−1^, with 16 scans, a resolution of 10 cm^−1^, a frequency of 2.2 Hz, and a DTGS detector. To prepare the garlic sample, it was peeled, sliced, vacuum freeze-dried, pulverized, and filtered through a 100-mesh sieve. A suitable amount of potassium bromide powder was ground into powder in an agate mortar, and then a small amount of powder was placed into a pellet press and pressed with a pressure of 10 MPa to form approximately 2 mm thick transparent sheets, followed by background scanning. To create the sample, approximately 1 mg of garlic sample powder was mixed evenly with potassium bromide at a ratio of 1:100 (m:m), pressed into pellets, and then scanned.

A UV-Vis spectrophotometer (model UV-5500, manufactured by Shanghai Yuanxi Instrument Co., Ltd., Shanghai, China) equipped with a GL-D2T-V01 UV-enhanced broadband deuterium-tungsten lamp and quartz cuvettes was employed for precise measurements. The cuvettes had an outer diameter of 12.4 × 12.4 × 45 mm, 10 mm light path, and 3.5 mL capacity. The spectrophotometer had a scanning wavelength range of 190~700 nm and a spectral resolution of 1 nm. A total of 225 garlic specimens sourced from five distinct origins were carefully peeled and crushed. Each sample was mixed with deionized water at a material-to-liquid ratio of 1:4 (m:V, g/mL). The mixture was then subjected to centrifugation at 10,000 r/min for 20 min. Finally, the supernatant was scanned for wavelengths using water as a reference solution.

### 2.3. Spectral Pretreatment

The spectral data of all garlic samples were randomly divided into a training set (70% of the data) and a test set (30% of the data). Prior to constructing the classification model, three commonly used spectral preprocessing methods were applied to preprocess the ultraviolet spectral data of garlic along with the mid-infrared spectral data. These methods are MSC, SG, and SNV. High-dimensional data contain a significant amount of redundant information that can obscure key relationships, leading to an increase in computational workload and reducing the reliability and stability of decision-making [19]. Therefore, to ensure data validity, characteristic wavelengths were selected for ultraviolet and mid-infrared spectroscopy using GA to find the optimal global solution.

### 2.4. Data Fusion

In this experiment, LLDF was employed to concatenate the mid-infrared and ultraviolet spectral data end-to-end, forming a novel fused spectral dataset. The selection of LLDF stems from its efficacy in effectively preserving original data information, ensuring consistent data formatting, and offering nuanced information unattainable by other fusion levels. This strategy enhances data accuracy and resolves issues of missing, erroneous, or redundant data [20]. Hence, LLDF was chosen to process the spectral data.

### 2.5. Modeling of Origin Classification

Spectral data from garlic samples of different origins were analyzed using Python 3.9. The data were pre-processed, and four classification methods, namely XGBoost, SVC, RF, and ANN, were applied to differentiate the spectral differences among the samples. Chemometrics techniques and machine learning algorithms were used to extract the spectral variances among the samples, which helped in identifying the origins of the garlic.

## 3. Results and Discussion

### 3.1. Spectral Analysis

**Figure 2:**
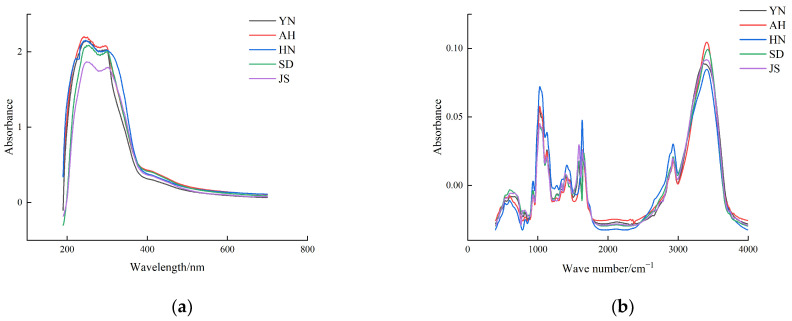
(**a**) Average wave number of ultraviolet spectral data; (**b**) average wavelength of mid-infrared spectral data.

Figure 2 shows the average ultraviolet spectral data and average mid-infrared spectral data curves of garlic from five different origins. Despite the similar profiles exhibited by the ultraviolet and mid-infrared spectral data curves of garlic samples from different origins, variations in absorbance at different wavelengths indicate differences in chemical component content among garlic from the five origins, which can be used to classify and trace the origin of garlic. The appearance of absorption peaks indicates the presence of specific chemical substances in the samples, with peak height representing the content of certain chemical substances [21,22]. In the ultraviolet spectral data, slight differences in absorbance of absorption peaks occur around 200–300 nm, particularly near 250 nm, which can be attributed to the π-π* electron transitions of the C=S bonding in garlic’s organosulfur compounds. This results in disparities in absorption peaks within the 200 nm to 300 nm range [23]. On the other hand, slight variations in absorption peaks in the mid-infrared spectra data from 1000–1500 cm^−1^ may stem from stretching vibrations of C-O bonds associated with organic lipid molecules present in garlic [24,25]. Peaks near 2900 cm^−1^ usually correspond to symmetric and asymmetric stretching vibrations of C-H bonds, reflecting the abundance of carbohydrates in garlic. The absorption peak near 3400 cm^−1^ typically corresponds to O-H bond stretching vibrations, potentially involving alcohols, phenols, or water molecules [26].

### 3.2. Spectral Preprocessing

The mid-infrared and ultraviolet spectroscopy data of garlic exhibit significant band overlap and broad peaks, accompanied by limited analytical information and excessive background noise, posing challenges in constructing accurate classification models. Combining spectral data preprocessing with machine learning techniques can enhance the accuracy of classification models. Ding et al. [27] utilized near-infrared spectroscopy to acquire spectral data of Huangshan Maofeng tea samples. They applied the SG algorithm for data smoothing and PCA for dimensionality reduction of the smoothed spectral data. Particle Swarm Algorithm (PSO) and Comprehensive Learning Particle Swarm Algorithm (CLPSO) were employed to optimize the penalty factor c and kernel function parameter g in the Support Vector Machines (SVM) model. The experimental results demonstrated that the CLPSO-SVM method achieved the highest classification performance, with a classification accuracy of 99.17%. Based on these findings, this study initially employed three preprocessing methods, including MSC, SG, and SNV, to extract the complete spectral structure of the signals. Subsequently, the three preprocessed spectral datasets were used as input variables to evaluate four classification models: XGBoost, SVC, RF, and ANN, for predicting the origin of garlic.

The results are presented in Table 1. For the ultraviolet spectral dataset, the accuracy of the SVC model in predicting the origin of garlic on the test set increased from 87.31% to 100%, 92.41%, and 100% after preprocessing with SG, MSC, and SNV, respectively. Similarly, the RF model’s accuracy in predicting the origin of garlic improved from 89.44% to 92.46%, 94.44%, and 91.42% on the test set after preprocessing with SG, MSC, and SNV, respectively. Although the mid-infrared spectral data model exhibited notable variations in performance across different data preprocessing algorithms within its test set, the most striking result was achieved after applying SNV preprocessing, which yielded an impressive accuracy of 94% for predicting the origin of garlic using the SVC model. By contrast, the accuracy rates obtained after SG and MSC preprocessing were significantly lower, at 40.00% and 19.31%, respectively. On the RF model, the accuracy for predicting the origin of garlic after SNV, SG, and MSC processing increased from 94.67% to 96.00%, 95.44%, and 97.59%, respectively.

After data preprocessing, four models were tested for their training and test set classification accuracy. The SNV method exhibited consistently stable performance. In the ultraviolet spectral dataset, the four models achieved accuracies of over 91% for predicting the origin of garlic following SNV data preprocessing. For the mid-infrared spectral dataset, the test set accuracies of SVC, RF, ANN, and XGBoost models in predicting the origin of garlic improved from 40%, 94.67%, 18.66%, and 90.67% to 94%, 96.00%, 84.00%, and 90.67%, respectively, after SNV data preprocessing. These findings indicate that SNV data preprocessing consistently produces more reliable model accuracy when compared to other preprocessing methods, making it the most suitable approach for further analysis.

### 3.3. Extraction of Characteristics

After preprocessing the ultraviolet and mid-infrared spectroscopy data, four classification models were established for garlic origin prediction. However, their accuracy was found to be suboptimal. To improve the accuracy, the GA for Feature Variable Selection was employed to extract wavelength features associated with the original spectra from both mid-infrared and ultraviolet spectral data. Table 2 shows that compared to the SVC model constructed on the original data, the accuracy of SG-GA-SVC based on mid-infrared spectral data decreased, while MSC-GA-SVC’s accuracy increased for garlic origin prediction. The four models displayed varying changes in garlic origin prediction accuracy across different preprocessing methods for ultraviolet spectroscopy. As shown in Table 2, the SVC model accuracy slightly decreased after SG and SNV preprocessing, while it slightly increased after MSC-GA preprocessing compared to MSC preprocessing.

Based on the findings presented in Table 2, it can be observed that the accuracy of both RF and SVC models improved in mid-infrared spectral data after SNV-GA preprocessing. However, the accuracy of ANN and XGBoost models decreased by 4% and 1.34% respectively. In the case of ultraviolet spectroscopy data, apart from the SVC model, which demonstrated a decrease in accuracy following GA-based feature extraction, the other three models showed improvements compared to their previous states.

In summary, the results suggest that the SNV-GA method effectively processed spectral data and produced consistent machine learning model performance. The observed consistency was presumably attributed to GA’s capability to effectively select and retain valuable features from preceding generations, thereby facilitating the search for an optimal solution to a given problem. Through multiple iterations, a collection of candidate sets were formed, ultimately leading to the discovery of the global optimal solution within this collection [28].

Spectral data features can be extracted more efficiently while retaining the original spectral features. The feature variables extracted by GA were combined with four classification models to assess their classification performance. Zheng et al. [29] proposed a BP neural network based on GA optimization for coal mine dust wettability identification. They compared it with a particle swarm optimization (PSO) extreme learning machine (ELM) algorithm. Results showed that the GA-BP model achieved the highest accuracy of 96.6% in discriminating coal mine dust wettability, followed by PSO-ELM, ELM, and BP models. Ge et al. [30] collected 114 samples of Taiping Monkey Kui green tea from four production areas, establishing SNV-ELM and SNV-GA-ELM models based on the combination of near-infrared spectroscopy and chemometrics to accurately identify green teas from specific geographic origins. The ELM model combined with SNV preprocessing achieved an accuracy of 93.07%, while the ELM model after SNV preprocessing combined with GA feature variables achieved an accuracy of 95.35% for the test set. The results demonstrated an increase in accuracy after the extraction of feature variables using GA.

### 3.4. Spectral Data Fusion

A fusion model that combines ultraviolet and mid-infrared spectral data was developed to improve the accuracy and stability of garlic origin prediction, aiming to obtain more comprehensive information about garlic samples. The SNV-GA method was identified as the optimal preprocessing technique to ensure stability in garlic origin prediction models. To develop the fusion of ultraviolet and mid-infrared spectral data, the SNV-GA approach was used for preprocessing and modeling. As demonstrated in Table 3, the fused spectral data achieved remarkable results in garlic origin prediction, boasting a perfect 100% accuracy across all four algorithmic models, including SVC, RF, ANN, and XGBoost. This suggests that the fused spectral model’s accuracy surpasses individual ultraviolet or mid-infrared spectral models after combining ultraviolet and mid-infrared spectral data.

In a previous study, Mariana K. et al. [31] used a combination of Fourier transform near-infrared and mid-infrared spectra to conduct LLDF. They developed a partial least squares-discriminant analysis (PLS-DA) model based on individual spectra and the LLDF spectroscopy. Their results showed that the discriminant model achieved higher accuracy in predicting test samples when compared to the individual models, with an accuracy of 94% or higher. Similarly, Jiang et al. [32] studied the use of near-infrared spectroscopy and hyperspectral imaging (HSI) data to detect adulteration in Ganoderma lucidum spore powder (GLSP). They found that near-infrared spectroscopy performed better than the HSI technique in identifying adulteration and predicting adulteration levels in GLSP when considering only a single spectral technique. Nevertheless, the introduction of a data fusion strategy rendered the MLDF approach highly effective in identifying adulteration, as evidenced by its impeccable performance with 100% accuracy, precision, recall, and F1 score at the random frog (RF) level.

### 3.5. Model Evaluation Metrics

True positive (TP), true negative (TN), false positive (FP), and false negative (FN) classifications are pivotal for method or model evaluation. TP denotes correctly identified positive instances, TN signifies accurately identified negative instances, FP indicates erroneously labeled negative instances as positive, and FN represents mistakenly classified positive instances as negative.

The classification metrics of TP, TN, FP, and FN are essential components in assessing the performance of methods or models. These metrics are directly related to the confusion matrix, a visual representation of the performance of a classification model. In the confusion matrix, TP, TN, FP, and FN are represented in different quadrants, providing a clear overview of the model’s ability to correctly classify instances. Let us delve deeper into the significance of these metrics and their interpretation within the context of the confusion matrix.

### 3.6. Confusion Matrix

**Figure 3:**
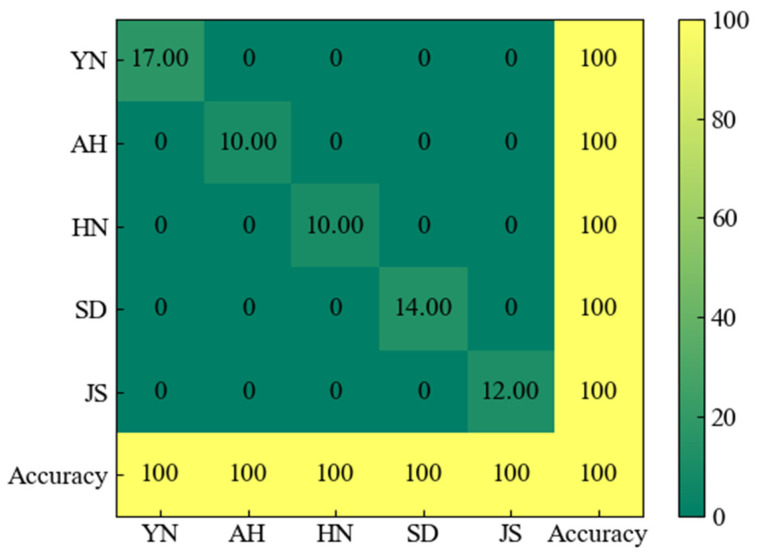
Confusion matrix of the four models under SNV-GA treatment.

The confusion matrix serves as an indispensable tool for assessing the classification accuracy of a given classifier. It effectively illustrates the correspondence between the actual characteristics of sample data and the classification prediction outcomes in a matrix format, providing a comprehensive view of the classifier’s performance. The rows of the matrix represent the predicted categories, while the columns represent the true categories. The cells on the diagonal indicate correctly categorized observations. The confusion matrix provides a clear overview of the number of correct and incorrect predictions made by the model. In Figure 3’s confusion matrix, it is clear that the number of Yunnan samples predicted among the actual Yunnan samples is 17. Moreover, it is noteworthy that there are no predicted samples available for the regions of Anhui, Henan, Shandong, and Jiangsu. This indicates a 100% accuracy in predicting purple-skinned garlic of Yunnan origin. Similarly, the prediction accuracy for the remaining four origins is also 100%, with no prediction errors found in the confusion matrix. After SNV-GA preprocessing, the four models achieved 100% accuracy on the test set, resulting in identical confusion matrices. Furthermore, after spectral data fusion, the four models achieved 100% accuracy on the test set for purple garlic from all five origins, indicating a significant improvement in model accuracy. This suggests that fused data can further enhance the accuracy of the models.

## 4. Conclusions

In this study, a dependable technique was effectively developed for the swift determination of garlic origin by amalgamating UV and mid-infrared spectra, as well as their fusion with low-level data. It was shown that the accuracy of the fusion spectra on the test sets of XGBoost, SVC, RF, and ANN models was 100%, whereas single spectra models exhibited a lower accuracy. Therefore, fusion spectra demonstrate more consistent accuracy than single spectral models. This study provides an effective technological approach for the identification of the origin of agricultural products and holds the potential for widespread application. In the future, the potential of this method can be further explored for use in other agricultural products, and it can be integrated with innovative technological tools like blockchain and IoT to develop a more extensive origin traceability system. The newly established system in this study can comprehensively ensure product quality and safety, thereby fostering greater trust among consumers.

## Figures and Tables

**Table 1:** Effect of preprocessing and algorithms on the classification accuracy of spectral data models.

Model	Preprocessing	Ultraviolet Spectrum		Mid-Infrared Spectrum	
		Train (%)	Test (%)	Train (%)	Test (%)
SVC	None	100	87.31	100	40.00
	SG	100	100	100	40.00
	MSC	100	92.41	100	19.31
	SNV	100	100	100	94
RF	None	100	89.44	100	94.67
	SG	100	92.46	100	95.44
	MSC	100	94.44	100	97.59
	SNV	100	91.42	100	96.00
ANN	None	100	93.45	26.84	18.66
	SG	100	100	26.84	18.66
	MSC	100	97.32	26.84	18.66
	SNV	100	97.65	92.44	84.00
XGboost	None	100	92.67	100	90.67
	SG	96.32	93.29	100	85.33
	MSC	100	95.34	100	91.43
	SNV	100	93.52	100	90.67

**Table 2:** Accuracy of model for classification of garlic origin after GA extracted features from UV and mid-infrared spectra.

Model	Preprocessing	Ultraviolet Spectrum		Mid-Infrared Spectrum	
		Train (%)	Test (%)	Train (%)	Test (%)
SVC	None	100	87.31	100	40.00
	SG-GA	100	96.05	100	26.66
	MSC-GA	100	96.68	100	28.45
	SNV-GA	100	98.54	100	97.33
RF	None	100	89.44	100	94.67
	SG-GA	100	98.56	100	97.34
	MSC-GA	100	93.47	100	94.67
	SNV-GA	100	99.16	100	97.34
ANN	None	100	93.45	26.84	18.66
	SG-GA	100	96.54	26.74	18.43
	MSC-GA	97.62	95.65	26.74	18.66
	SNV-GA	100	99.73	87.28	80.00
XGBoost	None	100	92.67	100	90.67
	SG-GA	100	97.92	100	90.56
	MSC-GA	100	96.38	100	85.34
	SNV-GA	100	97.41	100	89.33

**Table 3:** Spectral data fusion modeling applied to the classification prediction of garlic from different origins.

Model	Preprocessing	Fusion Spectrum	
		Train (%)	Test (%)
SVC	SNV-GA	100	100
RF	SNV-GA	100	100
ANN	SNV-GA	100	100
XGBoost	SNV-GA	100	100

## Data Availability

The original contributions presented in the study are included in the article, further inquiries can be directed to the corresponding author.
